# Working conditions and tuberculosis mortality in England and Wales, 1890–1912: a retrospective analysis of routinely collected data

**DOI:** 10.1186/s12879-016-1509-z

**Published:** 2016-05-20

**Authors:** Charlotte Jackson, Joanna H. Mostowy, Helen R. Stagg, Ibrahim Abubakar, Nick Andrews, Tom A. Yates

**Affiliations:** Centre for Infectious Disease Epidemiology, Research Department of Infection and Population Health, University College London, 4th Floor Mortimer Market, off Capper Street, London, WC1E 6JB UK; MRC Clinical Trials Unit at University College London, Aviation House, 125 Kingsway, London, WC2B 6NH UK; Tuberculosis Section, Public Health England, 61 Colindale Ave, London, NW9 5EQ UK; Statistics, Modelling and Economics Department, Public Health England, 61 Colindale Ave, London, NW9 5EQ UK

**Keywords:** Epidemiology, Occupation, Historical data

## Abstract

**Background:**

Modelling studies suggest that workplaces may be important sites of *Mycobacterium tuberculosis* transmission in high burden countries today. Contemporary data on tuberculosis by occupation from these settings are scarce. However, historical data on tuberculosis risk in different occupations are available and may provide insight into workplace transmission. We aimed to ascertain whether, in a high burden setting, individuals working in crowded indoor environments (exposed) had greater tuberculosis mortality than individuals employed elsewhere (unexposed).

**Methods:**

The Registrar General’s Decennial Supplements from 1890–2, 1900–2 and 1910–2 contain data on mortality from tuberculosis by occupation for men in England and Wales. In these data, the association between occupational exposure to crowded indoor environments and tuberculosis mortality was assessed using an overdispersed Poisson regression model adjusting for socioeconomic position, age and decade.

**Results:**

There were 23,962 deaths from tuberculosis during 14.8 million person-years of follow-up among men working in exposed occupations and 28,483 during 19.9 million person-years of follow-up among men working in unexposed occupations. We were unable to categorise a large number of occupations as exposed or unexposed. The adjusted rate ratio for death from tuberculosis was 1.34 (95 % confidence interval 1.26–1.43) comparing men working in exposed occupations to those in unexposed occupations.

**Conclusions:**

Tuberculosis mortality in England and Wales at the turn of the 20th century was associated with occupational exposure to crowded indoor environments. The association between working conditions and TB in contemporary high burden settings requires further study.

**Electronic supplementary material:**

The online version of this article (doi:10.1186/s12879-016-1509-z) contains supplementary material, which is available to authorized users.

## Background

Tuberculosis (TB) remains a major threat to human health, with an estimated 9.6 million incident cases globally in 2014 [1]. Historically, the TB burden in what today are industrialised low burden countries was higher than that currently seen in high burden countries, with mortality in some areas reaching 1000 per 100,000 per annum [2]. In 1901 in England and Wales, the annual risk of *Mycobacterium tuberculosis* (MTB) infection was estimated to have been 12 % [3]. This is considerably higher than in England and Wales today and somewhat higher than risks currently observed in settings with the highest TB burden, such as South Africa [4, 5], although contemporary risks may be underestimated [6]. The reasons for the decline in TB mortality in Europe before widespread implementation of effective biomedical interventions have been extensively debated [7–11]. Mathematical modelling suggests that declines after 1900 were due to reductions in new infections rather than reductions in the rate at which people progressed from infection to disease [12].

In high burden settings today, molecular epidemiology suggests that, at least in adults, the majority of MTB transmission occurs outside the home [13–16]. Some occupational environments are known to carry an increased risk of TB. For example, incidence is higher in healthcare workers [17] and in miners [18] than in the general population, due to healthcare settings concentrating infectious individuals and the high risk of silicosis (which dramatically increases susceptibility to TB), respectively. However, excess risk has also been noted in Peruvian public transport workers, suggesting that occupational exposure to large numbers of contacts in indoor congregate settings may carry an independent risk [19]. Mathematical models incorporating data on ventilation and social contact patterns from a high burden informal settlement near Cape Town have also suggested that workplaces and public transport may be where most transmission of MTB between adults occurs [20].

There are few comprehensive contemporary datasets on TB burden by occupation from high burden settings. We therefore looked for historical datasets from high burden settings with which to examine the association between occupation and TB. Supplements to the Registrar General’s reports from England and Wales provide cause-specific mortality statistics for men for the periods 1890–1892 [21], 1900–1902 [22] and 1910–1912 [23], stratified by occupation and age.

By utilising these data we aimed to better understand the relationship between TB mortality and occupational exposure to crowded indoor environments before the advent of vaccines and drugs with activity against TB. Specifically, we sought to determine whether men working in indoor spaces with many other people (exposed occupations) were more likely to die from TB than men who worked mainly outdoors or had less contact with others (non-exposed occupations).

Some of the results of this study have been previously reported at a conference [24] and a symposium.

## Methods

Data concerning male deaths from ‘phthisis’, an old name for pulmonary TB, during 1890–1892, 1900–1902 and 1910–1912 were extracted from scanned copies of the Registrar General’s Decennial Supplements, available online [21–23], into a Microsoft® Access 2010 database (Microsoft Corporation, Washington). These reports contain complete records of all recorded deaths in males during the respective time periods. JHM extracted numbers of deaths and person-years by age group for the 207 occupations which appeared in all three reports. A 10 % random sample was checked by CJ. Discrepancies were resolved by checking the original reports. In some cases, the reports presented data separately for several subgroups of the same occupation, e.g. categorised by geographical area. We combined such subsets prior to analysis.

Before analysis, JHM and TAY independently categorised occupations as exposed or unexposed, blinded to each other’s decisions (see Tables S1-S3 in Additional file 1). ‘Exposure’ was defined as working in an occupation likely to place workers in the same indoor public space as a substantial number of other people. This was a subjective assessment: factory work, mining, public-facing occupations and other occupations with comparable levels of exposure to crowded indoor public spaces were considered exposed. We considered enclosed public spaces, including buses and passenger trains, to be indoor public spaces. ‘Unexposed’ individuals worked in indoor spaces but with little contact with other people or worked outdoors. Occupations which could involve exposure to both types of environment, occupational groupings thought to contain both exposed and unexposed occupations, and occupations about which we had any doubts were placed in a third category. Discrepancies were resolved by consensus.

We adapted the socio-economic position (SEP) categorisation from the most recent of the Decennial Supplements [23], which classified occupations into eight socioeconomic groups. These are described in detail in the Registrar General’s Annual Report of 1911 [25]. Three SEP categories referred to specific occupational groups (miners, agricultural labourers and textile workers); we therefore re-assigned occupations within these groups to the remaining five SEP categories with reference to a later Registrar General classification [26]. This later classification assigned all occupations to one of five socioeconomic groups, group 1 corresponding to the ‘upper and middle classes’, group 3 to ‘skilled workmen’ and group 5 to ‘unskilled workmen’, with groups 2 and 4 defined in the original categorisation as ‘intermediate’ between groups 1 and 3 and groups 3 and 5, respectively.

We used the age groups given in the reports for 1890–92 and 1900–02: 15–19, 20–24, 25–34, 35–44, 45–54, 55–64 and ≥65 years. The dataset for 1910–12 divided the latter age group into 65–74 and ≥75 years but, for comparability with the earlier years, we combined these into a single age group. The dataset thus comprised counts of TB deaths and denominators, split by occupation, age group and period and with the occupation-level variables SEP and occupational category.

To estimate the relative change in rate of death from TB associated with working in an exposed occupation compared to an unexposed occupation, we initially used negative binomial regression to allow for extra-Poisson variation (overdispersion) in the data [27]. However, we found that this generated estimates of the unadjusted rate ratio that were inconsistent with the observed data (reasons for this are discussed below). We therefore used Poisson regression, incorporating a scale parameter to account for overdispersion, to estimate crude and adjusted rate ratios comparing TB mortality rates between occupational groups. We adjusted a priori for the potential confounders age, SEP and report decade. The baseline group for each of these confounders was taken as that with the largest denominator. For each covariate, p values were derived using likelihood ratio tests to compare the given model to the null model with the scale parameter constrained to equal that estimated in the model of interest.

While investigating the reasons for the discrepant results from the negative binomial model, we noted that the association between occupational category and TB mortality appeared to vary according to the number of men employed in each occupation. We therefore also present results of post hoc analyses stratified by tertile of denominator size.

We conducted several sensitivity analyses to assess the robustness of our conclusions. First, we fitted the crude and adjusted overdispersed Poisson models to a dataset excluding occupations likely to be at elevated risk of TB mortality due to risk factors other than working indoors with many other people: healthcare professionals, farmers (who may have contact with cattle) and occupations in which workers might be exposed to dust, such as silica [22] (Additional file 1: Table S4). Second, we excluded general labourers (the occupation contributing the largest number of person-years in the uncategorised group) from the analysis. This allowed us to assess the extent to which categorisation of this large group of men, who suffered high TB mortality, determined observed differences between groups. Third, we expanded the aggregated dataset into a line listing containing the equivalent individual level data. We conducted a random effects logistic regression analysis of the resulting dataset, overall and stratified by tertile of occupation size, to take account of the potential for clustering by occupation.

All analyses were conducted using Stata 14 (Stata Corporation, Texas).

The extracted dataset and our Stata code have been made available as supplementary materials to enable readers to re-run these analyses with alternative categorisations of occupations as ‘exposed’ or ‘unexposed’ (Additional files 2-4).

This study is based on publicly available, historical, aggregated (non-identifiable) data and therefore did not require ethical approval.

## Results

Where both JHM and TAY agreed that occupations were categorisable, there was agreement about exposure status in 55 of 57 instances (Cohen’s Kappa 0.92). The two disagreements and all professions considered uncategorisable by either JHM or TAY were placed in the uncategorised group.

**Fig 1:**
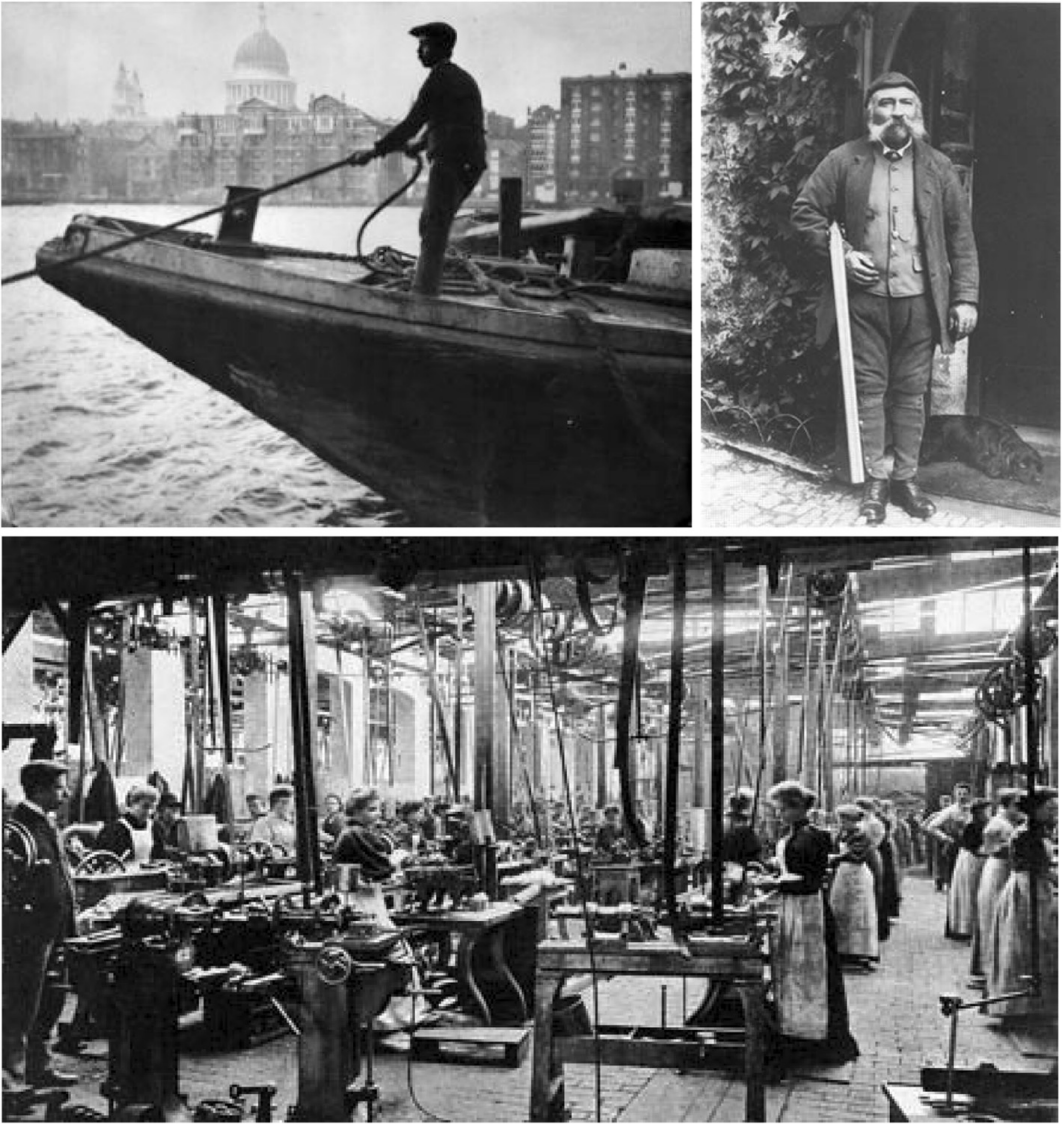
A London lighterman circa 1910 (top left) and, George Barnes, gamekeeper on the Dynevor estate, Wales, circa 1900 (both unexposed occupations), plus workers in a bicycle factory, Coventry, 1911 (an exposed occupation)

After combining subsets of the same occupation, the dataset contained information on 154 occupations. 37 occupations were categorised as exposed (occupations with extensive indoor contact), 16 as unexposed (occupations with little indoor contact) and 101 were placed in the third uncategorised group (Fig. 1 and Additional file 1: Tables S1-S3).

Amongst 646 numbers (numbers of deaths or person years of follow up time) extracted by two researchers, there were 11 discrepancies. All were found to be errors in the 10 % sample entered for checking and not in the original data extract. Subsequent data exploration identified seven further errors, which were corrected, and one omitted record, which was then entered.

A total of 82.3 million person years of data were available and 164,667 TB deaths recorded (Table 1). The largest professions were coal miners (exposed, 5.9 million person-years), farm labourers or farm servants (unexposed, 5.7 million person-years) and general labourers (uncategorised, 5.7 million person-years) (Additional file 1: Tables S1-S3). Overall, the greatest number of person-years was seen in the uncategorised occupations, which also had the largest number of deaths.

**Table 1:** Total numbers of deaths from ‘phthisis’ and denominators by occupational category, 1890–92, 1900–02 and 1910–12

Period	Exposed Occupations			Unexposed Occupations			Uncategorised Occupations		
	Number of deaths	Person years of follow up	Mortality rate per 100,000 per year	Number of deaths	Person years of follow up	Mortality rate per 100,000 per year	Number of deaths	Person years of follow up	Mortality rate per 100,000 per year
1890–1892	8003	3,778,203	211.8	11,921	6,612,453	180.3	35,883	12,692,445	282.7
1900–1902	6773	4,215,931	160.7	9484	6,968,242	136.1	36,543	15,457,624	236.4
1910–1912	9186	6,804,315	135.0	7078	6,278,787	112.7	39,796	19,541,255	203.7
Total	23,962	14,798,449	161.9	28,483	19,859,482	143.4	112,222	47,691,324	235.3

**Fig 2:**
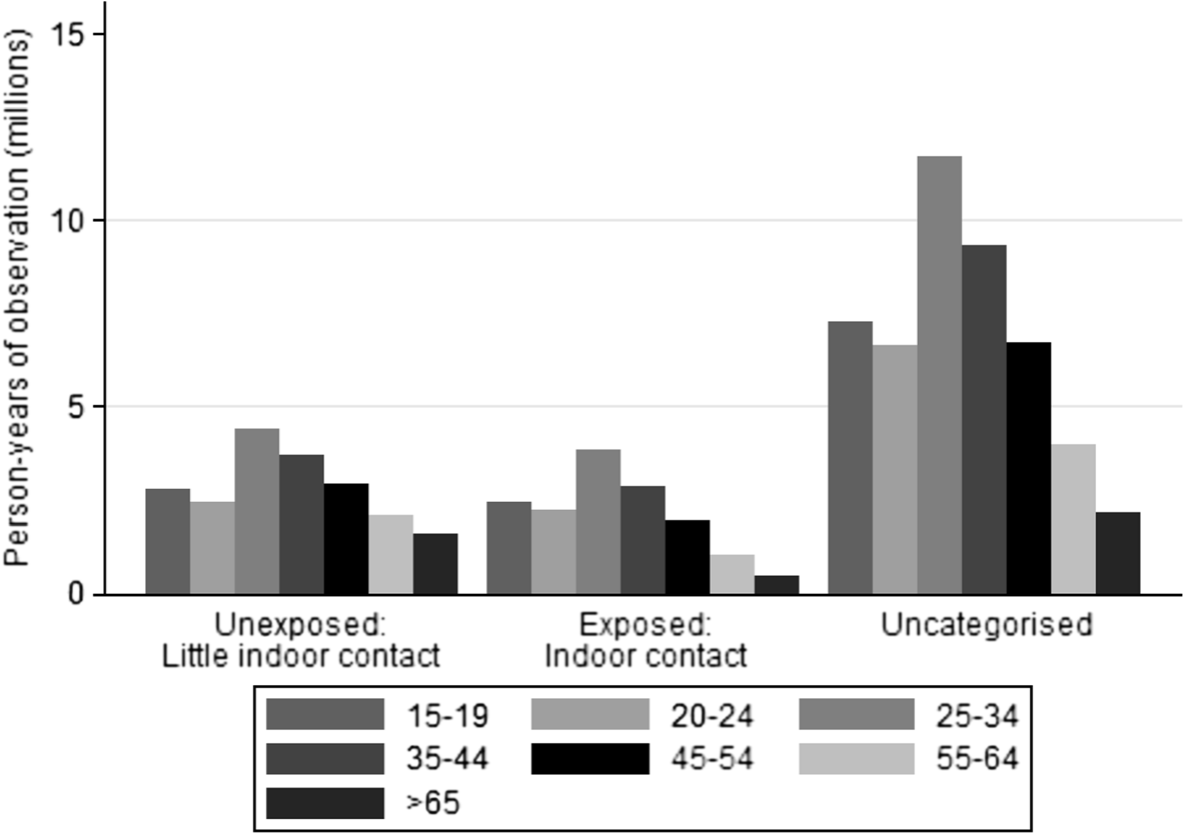
Person-years of observation (in millions) amongst men in each occupational category, by age group

**Fig 3:**
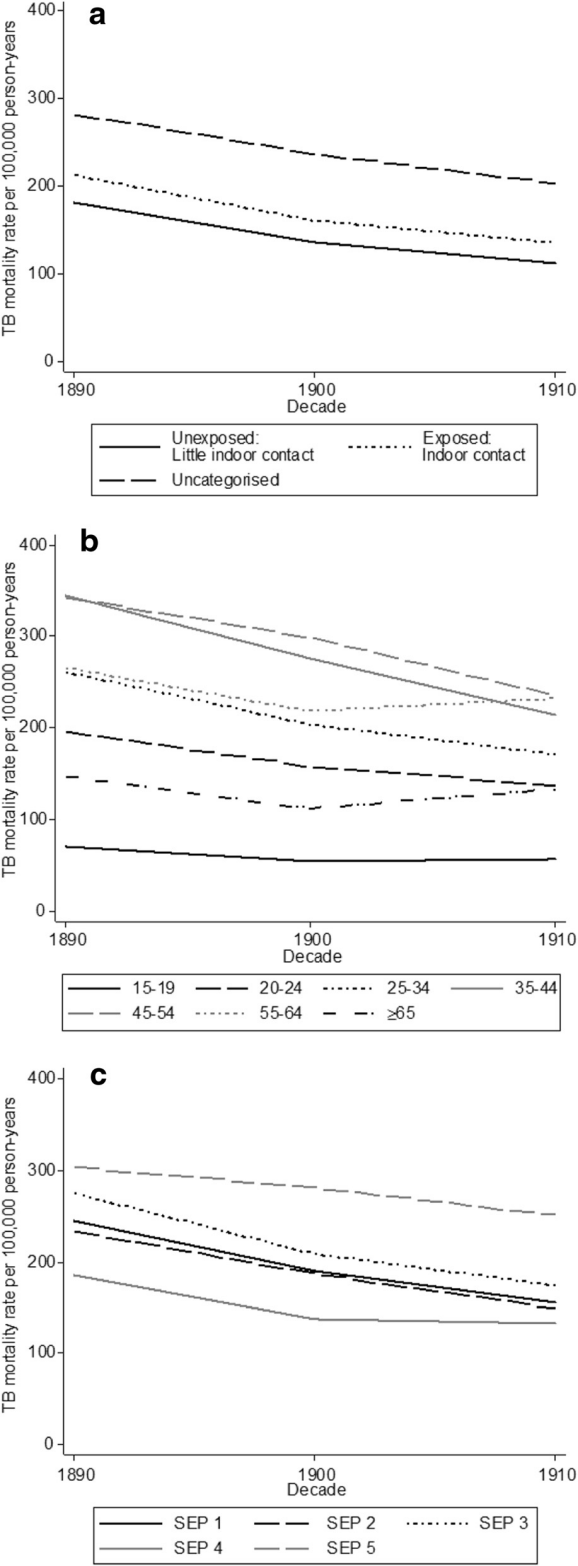
TB mortality rates over time (per 100,000 person-years), 1890–1912, by **a** occupational exposure to crowded indoor spaces **b** age group (in years) and **c** socioeconomic position (SEP, where group 1 is the highest position and group 5 the lowest)

Men aged 25–34 years were the largest group in all three occupational categories (Fig. 2). Crude mortality rates decreased over time in most age groups (Fig. 3b) and socio-economic categories (Fig. 3c). The highest crude TB mortality rates were among men aged 45–54 and 35–44 years. The youngest and the oldest men had the lowest mortality rates. Men with higher SEP generally had lower crude mortality rates compared to men with lower SEP, although the lowest rates occurred in SEP group 4 (which includes agricultural labourers, who had low TB mortality rates).

The crude mortality rate for TB was higher among men working in exposed occupations than in men working in unexposed occupations (162 versus 143 per 100,000 person-years, Table 1 and Fig. 3a). However, the highest crude mortality rates were observed in the third, uncategorised, group of occupations (235 per 100,000 person-years, Table 1). Crude mortality rates and the numbers of deaths per occupation are provided as supplementary data (Additional file 1: Tables S1-S3).

Both crude (Table 2) and adjusted (Table 3) Poisson regression models provided evidence that working in crowded indoor spaces was associated with an increase in mortality rates from TB compared to working indoors with little contact or in an outdoor occupation. After adjusting for age, SEP and decade, working in a crowded indoor environment was associated with a 34 % higher rate of TB mortality (rate ratio [RR] 1.34 [95 % CI 1.26–1.43]). Men in occupations which we were unable to categorise experienced higher TB mortality rates than those whose jobs involved extensive indoor contact, with an adjusted RR of 1.71 (95 % CI 1.62–1.79) compared to men working outdoors or with little indoor contact (Table 3). Excluding the uncategorised group had little effect on the estimated crude or adjusted RR for exposed men (1.13 [1.02–1.25] and 1.43 [1.33–1.54], respectively [Additional file 1: Table S5]).

**Table 2:** Crude associations of occupation category and key covariates with mortality from ‘phthisis’

Variable		Crude Rate Ratio (95 % CI)	p value
Occupation Category	Unexposed	Referent	<0.0001
	Exposed	1.13 (1.03–1.24)	
	Uncategorised	1.64 (1.53–1.76)	
Age in years	15–19	0.29 (0.26–0.32)	<0.0001
	20–24	0.78 (0.72–0.84)	
	25–34	Referent	
	35–44	1.30 (1.23–1.38)	
	45–54	1.38 (1.29–1.47)	
	55–64	1.15 (1.06–1.24)	
	>65	0.63 (0.56–0.72)	
Socioeconomic position	1 (highest)	1.25 (1.12–1.39)	<0.0001
	2	1.24 (1.14–1.36)	
	3	1.42 (1.32–1.53)	
	4	Referent	
	5 (lowest)	1.83 (1.71–1.97)	
Period	1890–1892	Referent	<0.0001
	1900–1902	0.82 (0.77–0.88)	
	1910–1912	0.71 (0.67–0.76)

**Table 3:** Adjusted associations between occupation category and mortality from ‘phthisis’. In sensitivity analysis, occupations at risk of TB for other reasons were excluded^a^

	Including all occupations		Excluding occupations at risk of TB for other reasons	
Occupation category	Adjusted rate ratio (95 % CI) ^b^	p-value	Adjusted rate ratio (95 % CI) ^b^	p-value
Unexposed	Referent	<0.0001	Referent	<0.0001
Exposed	1.34 (1.26–1.43)		1.46 (1.37–1.57)	
Uncategorised	1.71 (1.62–1.79)		1.59 (1.51–1.68)	

^a^Healthcare workers, farmers and occupations with dust exposure

^b^Analysis adjusted for age, socioeconomic position and decade

Sensitivity analysis excluding occupations at risk of TB for other reasons produced similar results (adjusted RR 1.46 [1.37–1.57] and 1.59 [1.51–1.68] for the exposed and uncategorised groups, respectively, compared to the unexposed (Table 3) or 1.64 [1.52–1.76] for the exposed if the uncategorised group was additionally excluded [Additional file 1: Table S6]). Excluding general labourers, the largest group of uncategorised workers, did not substantially alter estimates from the overdispersed Poisson model (adjusted RR 1.30 (95 % CI 1.23–1.39) and 1.62 (95 % CI 1.54–1.70) for exposed and uncategorised occupations, respectively, Additional file 1: Table S7).

Following post hoc stratification by tertile of population size, we found that the crude (but not the adjusted) RR comparing the exposed and unexposed groups, as estimated by overdispersed Poisson regression, decreased with increasing denominator (Additional file 1: Table S8). However, when high-risk occupations (as defined a priori) were excluded and tertiles re-calculated accordingly, this trend was less evident (Additional file 1: Table S9).

The adjusted odds ratios from random effects logistic regression of the individual level dataset were 1.69 (95 % CI 1.33–2.14) for the exposed group and 1.71 (95 % CI 1.39–2.11) for the uncategorised group (Additional file 1: Table S10). When stratified by tertile of denominator (Additional file 1: Table S10), neither crude nor adjusted odds ratio estimates from random effects logistic regression differed systematically from the RRs based on the original aggregated data (Additional file 1: Table S8).

As noted above, crude results from negative binomial regression were inconsistent with the raw data. Crude and adjusted estimates from negative binomial regression, including stratification by denominator size, are presented in the supplementary material (Additional file 1: Tables S11 and S12). These estimates were similar to those from the random effects logistic regression, but with narrower CIs.

## Discussion

We present evidence that working in a crowded indoor space was associated with a one-third increase in mortality rates from TB compared to working in other indoor spaces or working outdoors in an analysis adjusted for age, socioeconomic position and time period. TB was a leading cause of premature mortality in England and Wales over this period, as it is currently in high burden settings [1, 2]. The force of infection in England and Wales over this period was probably higher than anywhere today, except perhaps in some modern high burden urban communities [3–6]. Levels of poverty, inequality and overcrowding may be comparable, although the climate and social context are clearly very different. Other major differences include the HIV epidemic, mass BCG vaccination and the availability of effective TB treatment, plus plausibly differences in circulating MTB strains and host genetics.

The strengths of this analysis include the size of the dataset, its (theoretical) completeness in recording cause-specific mortality for all men during these periods and the detailed categorisation of occupation. However, the study has several limitations. TB during this period was generally diagnosed clinically rather than microbiologically or using x-ray and thus there may have been some misattribution of cause of death [28]. There may also have been a reluctance among clinicians and coroners to list TB as the cause of death, as TB was stigmatised and the diagnosis might prevent surviving family members accessing medical insurance [28]. Any such misclassification would likely be independent of working conditions and thus would bias our effect estimates towards the null. Mandatory notification of clinical TB cases was not introduced until 1912 [29] so would have very little impact on our analyses.

We used an old system to classify SEP. Whilst more detailed methods have since been developed to quantify the complex concept of SEP, modern classifications [30] would not necessarily be easily applicable to historical occupations. However, there may be some misclassification of SEP in our analysis, e.g. some occupation titles used in the dataset did not match exactly with those used in the SEP classification, and some occupational groups might include both skilled and less skilled workers which would be combined in the same SEP category.

At the turn of the twentieth century, only 32 % of females over the age of 10 years worked outside the home [31], largely women from low income households [32]. Of these, 44 % worked in ‘family, institutional or personal service’ and 56 % in industry (mostly in the clothing or textile industries) [31]. The data available to us were limited to males and thus may not be generalisable to females. However, it seems unlikely that any association between TB mortality and working in crowded indoor environments would differ substantially by gender.

Registered occupation was the last profession of the deceased men and we assumed that this was a valid proxy for exposure status at the time of acquisition of TB infection. However, men may have died from TB acquired whilst working in a different profession. It is possible, for example, that men might move into non-manual occupations on becoming symptomatic. The direction of this bias and thus its impact on the effect estimate would depend on whether such individuals were more likely to move into or out of exposed occupations. In the source data, numbers of deaths were taken from death registers while denominators were based on census returns [22], which has at least two implications for the analysis. First, men may have changed their jobs between the census and their death, leading to inaccurate denominators. Second, there may be discrepancies between the two data sources in reporting an individual’s occupation, meaning that numerators and/or denominators are incorrect [23].

The dataset contained no information on proximal risk factors for TB mortality, such as smoking, alcohol consumption, nutritional status, or comorbidities, nor did it contain data on exposure to TB in other settings, such as social venues or crowded accommodation. Whilst adjustment for SEP may have accounted for some of these differences, we cannot exclude the possibility that our results are influenced by confounding by such unmeasured factors. Specifically, we did not have data to adjust for urban versus rural residence. It seems likely that employment in exposed occupations was associated with urban residence. Should urban men have greater contact with TB outside work, smoke or drink more, be less well nourished, or be generally less healthy, this might, in part, explain the association we observed. Conceiving similar systematic differences as putative explanations for the high mortality in the uncategorised group is more challenging, given that this group contained such a heterogeneous set of occupations.

Although occupations were categorised independently by two researchers, the groupings made may be subject to misclassification. The large number of occupations that remained unclassified reflects the cautious approach adopted. Should occupations in the uncategorised group with high TB mortality have, truly, been unexposed then the effect would be more modest than estimated. However, the association was still observed when general labourers, the largest group of uncategorised workers, were excluded from the analysis, and when occupations at risk of TB for other reasons were dropped.

The inconsistency of the estimates from the negative binomial model with the observed data is interesting. Differences between negative binomial and quasi-Poisson models have also occasionally been noted in ecology, being attributed to the different weighting of data points in the two models: the negative binomial gives a similar weight to all observations with expected death counts above one whereas the quasi-Poisson model gives increasing weight to points with higher expected death counts [33]. The larger effect size seen with the negative binomial model can therefore be explained by the presence of some small but high risk exposed occupations, such as tin miners, lead miners and copper miners, which have greater influence on this model. This effect could also be seen when the quasi-Poisson model was fitted to data stratified by the population size where a greater effect was seen in those occupations with smaller denominators than those with larger denominators.

However, regardless of the model used and in all sensitivity analyses, we found evidence that working indoors in a high-contact environment was associated with an increased risk of dying from pulmonary TB.

## Conclusions

Our finding that occupational exposure to crowded indoor environments in England and Wales at the turn of the 20th Century was associated with TB mortality should prompt urgent assessment of whether similar associations can be observed in high burden settings today. Employment in certain occupations clearly predicts TB risk [17, 18]. A major contribution of exposure in the workplace to TB transmission, beyond specific high-risk occupations, is predicted by mathematical models [20]. Were this to be demonstrated empirically, infection control precautions, including low cost environmental modifications, might be deployed in these spaces to cut TB transmission risk [34–37] and case finding might be targeted at such workplaces [37].

## Ethics approval and consent to participate

As this was an analysis of publically available, aggregated and anonymised data, we did not seek ethics approval before undertaking these analyses.

## Availability of data and materials

This article has an online data supplement, containing additional tables. We have also made available the full dataset and Stata .do files for the analyses undertaken.

## Additional files

Additional file 1: Occupations and TB mortality Supplement. (DOCX 60 kb) Additional file 2: E&W historical TB mortality data. (CSV 191 kb) Additional file 3: Main analysis do file. (DOC 31 kb) Additional file 4: Random effects analysis overall and by tertile of denominator. (DOC 3 kb)

## Abbreviations

BCG: Bacillus Calmette–Guérin vaccine
HIV: Human Immunodeficiency Virus
MTB: Mycobacterium tuberculosis
RR: rate ratio
SEP: socio-economic position
TB: Tuberculosis
