# Supplementary material for: Working conditions and tuberculosis mortality in England and Wales, 1890–1912: a retrospective analysis of routinely collected data
Source: BMC Infect Dis. 2016 May 20;16:215. doi: 10.1186/s12879-016-1509-z (PMC4875674; doi:10.1186/s12879-016-1509-z)
Supplement: Additional file 1: — Occupations and TB mortality Supplement. (DOCX 60 kb) [file 12879_2016_1509_MOESM1_ESM.docx]

**Working conditions and tuberculosis mortality in England and Wales, 1890-1912: a retrospective analysis of routinely collected data**

Charlotte Jackson, Joanna H. Mostowy, Helen R. Stagg, Ibrahim Abubakar, Nick Andrews, Tom A. Yates

Online data supplement

Table S1. Exposed occupations (involving high levels of indoor contact), in descending order of mortality rate.

| **Occupation** | **Person-years** | **Number of deaths from phthisis** | **Crude mortality rate per 100,000 per year** |
| --- | --- | --- | --- |
| Tin miner | 68091 | 455 | 668.2 |
| Lead miner | 39072 | 179 | 458.1 |
| Copper miner | 5571 | 25 | 448.8 |
| Barmen | 86949 | 345 | 396.8 |
| Musician, music-master | 189324 | 657 | 347.0 |
| Potter; earthenware, &c. manufacturer | 320700 | 995 | 310.3 |
| Hairdresser | 289224 | 778 | 269.0 |
| Glass manufacture | 225423 | 606 | 268.8 |
| Waiters | 69933 | 187 | 267.4 |
| Tinplate goods makers | 59205 | 156 | 263.5 |
| Cotton, flax, linen, manufacture | 1057494 | 2527 | 239.0 |
| Silk, satin, crape, &c. manufacture | 98097 | 234 | 238.5 |
| Hosiery manufacture | 136533 | 321 | 235.1 |
| Nail, anchor, chain, and other iron and steel manufactures | 1257126 | 2874 | 228.6 |
| Stationery, envelope, cardboard box, &c. manufacture | 41172 | 92 | 223.5 |
| Carpet, rag, manufacture | 66867 | 147 | 219.8 |
| Lace manufacture | 118980 | 257 | 216.0 |
| Cycle makers | 82434 | 174 | 211.1 |
| Wool, worsted, manufacture | 750831 | 1538 | 204.8 |
| Nail, bolt, lock, key - makers | 54765 | 95 | 173.5 |
| Wool - sorting, carding & combing processes | 48762 | 84 | 172.3 |
| Cotton blow room hands | 28545 | 49 | 171.7 |
| Cotton stripers & grinders | 14940 | 25 | 167.3 |
| Straw plait, straw hat, straw bonnet - manufacture | 15078 | 23 | 152.5 |
| Iron and steel manufacture; iron goods makers | 862977 | 1314 | 152.3 |
| Ironstone miner | 103764 | 152 | 146.5 |
| Cotton manufacture | 642639 | 931 | 144.9 |
| Tallow, soap manufacture | 163437 | 235 | 143.8 |
| Paper manufacture | 127476 | 182 | 142.8 |
| Anchor, chain manufacture; blacksmith | 406050 | 574 | 141.4 |
| Schoolmaster, teacher | 537981 | 728 | 135.3 |
| Cycle and motor manufacture | 2532 | 3 | 118.5 |
| Coal miner | 5858274 | 6138 | 104.8 |
| Physician, surgeon, general practitioner | 197925 | 202 | 102.1 |
| Coach, motor, railway carriage, tram car, &c. - makers | 365178 | 346 | 94.7 |
| Iron - miners, quarriers | 67887 | 60 | 88.4 |
| Clergyman, priest, minister | 337213 | 274 | 81.3 |

Table S2. Unexposed occupations (involving little indoor contact) in descending order of mortality rate.

| **Occupation** | **Person-years** | **Number of deaths from phthisis** | **Crude mortality rate per 100,000 per year** |
| --- | --- | --- | --- |
| Dock labourer, wharf labourer | 745317 | 2669 | 358.1 |
| Bricklayer, mason, builder | 2187492 | 5261 | 240.5 |
| Bargeman, lighterman, waterman | 264381 | 516 | 195.2 |
| Carman, carrier, &c | 2210298 | 4256 | 192.6 |
| Artist, engraver, sculptor, architect | 180415 | 337 | 186.8 |
| Fisherman | 221730 | 302 | 136.2 |
| Labourer, &c., in agricultural districts | 2019072 | 2676 | 132.5 |
| Engine driver, stoker, fireman (not railway, marine, nor agricultural) | 899688 | 1068 | 118.7 |
| Coke burner | 26472 | 29 | 109.5 |
| Gardener, nurseryman, seedsman | 1907535 | 2080 | 109.0 |
| Farm labourer, farm servant | 5646978 | 6112 | 108.2 |
| Railway engine driver, stoker | 533208 | 490 | 91.9 |
| Farmer, grazier, farmer's son, &c | 2624127 | 2365 | 90.1 |
| Motor car, motor van drivers | 129204 | 116 | 89.8 |
| Gamekeeper | 103227 | 82 | 79.4 |
| Platelayers, gangers, packers | 160338 | 124 | 77.3 |

Table s3. Uncategorised occupations in descending order of mortality rate.

| **Occupation** | **Person-years** | **Number of deaths from phthisis** | **Crude mortality rate per 100,000 per year** |
| --- | --- | --- | --- |
| Costermonger, hawker, &c | 434667 | 2276 | 523.6 |
| Inn, hotel-servant | 276330 | 1253 | 453.4 |
| Tool, scissors, file, saw, needle, maker | 391872 | 1470 | 375.1 |
| General labourer | 5719986 | 20616 | 360.4 |
| Chimney sweep, soot merchant | 44214 | 155 | 350.6 |
| Slate - quarriers, workers | 39828 | 131 | 328.9 |
| Gunsmith | 79140 | 259 | 327.3 |
| Inn-keeper, publican; spirit, wine, beer, dealer | 761346 | 2463 | 323.5 |
| Bookbinder | 108987 | 349 | 320.2 |
| Seaman, &c, merchant service | 933986 | 2974 | 318.4 |
| Patent fuel manufacture | 3459 | 11 | 318.0 |
| Brush, broom maker; hair, bristle worker | 56058 | 177 | 315.7 |
| Printer | 846696 | 2632 | 310.9 |
| Stone getters, dressers; masons | 350007 | 1065 | 304.3 |
| Tobacconist | 125352 | 375 | 299.2 |
| Shoemaker | 1733493 | 5180 | 298.8 |
| Hatter | 144618 | 432 | 298.7 |
| Brewer | 245667 | 708 | 288.2 |
| Stone, slate, quarrier | 359598 | 1023 | 284.5 |
| Tailor | 1207362 | 3319 | 274.9 |
| General shopkeeper | 278283 | 749 | 269.2 |
| Inn, hotel, &c. – servants | 255330 | 687 | 269.1 |
| Brass, bronze-manufacturers, founders, finishers, workers | 269047 | 719 | 267.2 |
| Wood turner, cooper, &c | 344682 | 914 | 265.2 |
| Cabinet maker, &c | 788046 | 2074 | 263.2 |
| Currier, &c | 213162 | 547 | 256.6 |
| Tobacco manufacture | 23841 | 61 | 255.9 |
| Publisher, bookseller, stationer, &c | 215928 | 552 | 255.6 |
| Locksmith, bellhanger, gasfitter | 125637 | 321 | 255.5 |
| Coach and cab service, groom, &c | 1693758 | 4257 | 251.3 |
| Copper, tin, zinc, lead, brass, &c., worker and dealer | 838047 | 2094 | 249.9 |
| Coal heaver | 230313 | 562 | 244.0 |
| Commercial clerk, insurance service | 1813653 | 4332 | 238.9 |
| Slater, tiler | 74808 | 177 | 236.6 |
| Law clerk | 279468 | 657 | 235.1 |
| Watch, clock, phil. inst., maker, jeweller, &c | 640563 | 1505 | 234.9 |
| Furrier, skinner | 25812 | 60 | 232.5 |
| Plumber, painter, glazier | 1422954 | 3302 | 232.1 |
| Wool, silk, cotton, &c.; dyer, printer, &c | 460947 | 1055 | 228.9 |
| Paperhanger, plasterer, whitewasher | 315297 | 718 | 227.7 |
| Draper, Manchester warehouseman | 583953 | 1326 | 227.1 |
| Photographer | 36297 | 82 | 225.9 |
| Piano, organ – makers | 42852 | 94 | 219.4 |
| Insurance agents | 162756 | 350 | 215.0 |
| Beer bottlers; cellarmen | 44232 | 95 | 214.8 |
| Brass, bronze - manufacture; brassfounders | 36804 | 79 | 214.7 |
| Messenger, porter, &c. (not railway nor government) | 1053783 | 2250 | 213.5 |
| Railway labourers | 78636 | 166 | 211.1 |
| Copper manufacture; copper workers; coppersmiths | 37665 | 79 | 209.7 |
| Commercial clerks | 1079148 | 2227 | 206.4 |
| Rope, twine, cord-maker | 74280 | 148 | 199.2 |
| Painters, decorators | 570453 | 1135 | 199.0 |
| Commercial traveller | 587040 | 1163 | 198.1 |
| Railway official, clerk | 592272 | 1172 | 197.9 |
| Wire - drawers makers, workers, weavers | 57771 | 113 | 195.6 |
| India rubber, gutta percha worker; waterproof goods maker | 85056 | 164 | 192.8 |
| Gas works service | 400761 | 765 | 190.9 |
| Coach, carriage-maker | 269190 | 507 | 188.3 |
| Blacksmith, whitesmith | 812868 | 1513 | 186.1 |
| Carpenter, joiner | 2101740 | 3911 | 186.1 |
| Fruiterer, greengrocer | 364134 | 670 | 184.0 |
| Engine, machine, boiler, maker, fitter; millwright | 2678403 | 4892 | 182.6 |
| Dealers in paper, prints, books & stationery | 124857 | 222 | 177.8 |
| Corn miller | 133497 | 235 | 176.0 |
| Saddler, harness maker | 309701 | 541 | 174.7 |
| Chemist, druggist | 219285 | 383 | 174.7 |
| Fishmonger, poulterer | 272442 | 472 | 173.2 |
| Tramway service | 177794 | 308 | 173.2 |
| Baker, confectioner | 934545 | 1615 | 172.8 |
| Domestic indoor servant | 454572 | 785 | 172.7 |
| Butcher | 973707 | 1648 | 169.3 |
| Scientific weighing and general instrument makers | 70254 | 117 | 166.5 |
| Bread, biscuit, cake, &c. – makers | 239880 | 378 | 157.6 |
| Shipwright, &c. (wood and iron) | 438711 | 665 | 151.6 |
| Wheelwright | 240443 | 358 | 148.9 |
| Tanner, fellmonger | 90912 | 135 | 148.5 |
| Railway platelayer, railway road, clay, sand, &c., labourer | 803640 | 1167 | 145.2 |
| Railway guard, porter, pointsman, &c | 1110045 | 1575 | 141.9 |
| Manufacturing chemist | 228132 | 320 | 140.3 |
| Grocer, &c | 1328613 | 1853 | 139.5 |
| Sawyer | 283749 | 394 | 138.9 |
| Ironmonger | 225348 | 312 | 138.5 |
| Millers, cereal food manufacture | 73620 | 101 | 137.2 |
| Civil service (messengers, &c.), including retired | 218889 | 297 | 135.7 |
| Milkseller, cheesemonger, &c | 385446 | 518 | 134.4 |
| Maltster | 84756 | 112 | 132.1 |
| Lithographer; copper and steel plate printer | 38238 | 49 | 128.1 |
| Electrical apparatus makers, electricians, electrical fitters | 251310 | 318 | 126.5 |
| Coal, coke – merchant, dealer | 235896 | 290 | 122.9 |
| Civil service (officers and clerks), including retired | 225699 | 277 | 122.7 |
| Barrister, solicitor | 191934 | 235 | 122.4 |
| Furniture dealers | 72468 | 87 | 120.1 |
| Shipbuilding | 322626 | 378 | 117.2 |
| Plaster, cement – manufacture | 29919 | 35 | 117.0 |
| Provision dealers | 56814 | 59 | 103.8 |
| Brick, tile, maker, burner | 432972 | 430 | 99.3 |
| Builders | 132393 | 126 | 95.2 |
| Insurance - officials, clerks | 127716 | 121 | 94.7 |
| Corn, flour, seed - merchants, dealers | 55704 | 48 | 86.2 |
| Bankers, bank officials, clerks | 128043 | 110 | 85.9 |
| Electricity supply | 44418 | 36 | 81.0 |

Table S4. High risk occupations excluded in sensitivity analysis.

|  | **Healthcare professionals** | **Farmers** | **Occupations exposed to silica dust** |
| --- | --- | --- | --- |
| Exposed occupations | Physician, surgeon, general practitioner |  | Coal miner  Copper miner  Ironstone miner  Lead miner  Tin miner  Iron miners, quarriers  Potter, earthenware, manufacturer |
| Unexposed occupations |  | Farm labourer, farm servant  Farmer, grazier, farmer's son, &c | Coke burner |
| Uncategorised occupations |  |  | Tool, scissors, file, saw, needle makers  Cutlers  Stone getters, dressers, masons |

Table S5. Crude and adjusted* associations between occupation category and mortality from ‘phthisis’ in Poisson regression (with overdispersion), excluding uncategorised occupations.

| Variable | | Crude Rate Ratio (95% CI) | p-value | Adjusted Rate Ratio (95% CI) | p-value |
| --- | --- | --- | --- | --- | --- |
| Occupation Category | Unexposed | Reference | <0.0001 | Reference | <0.0001 |
|  | Exposed | 1.13 (1.02-1.25]) |  | 1.43 (1.33-1.54) |  |

* Adjusted for age group, year and socioeconomic position.

RR = rate ratio; CI = confidence interval

Table S6. Crude and adjusted* associations between occupation category and mortality from ‘phthisis’ in Poisson regression (with overdispersion) excluding the uncategorised occupations and occupations at risk of TB for other reasons.

| Variable | | Crude Rate Ratio (95% CI) | p-value | Adjusted Rate Ratio (95% CI) | p-value |
| --- | --- | --- | --- | --- | --- |
| Occupation Category | Unexposed | Reference | 0.024 | Reference | <0.0001 |
|  | Exposed | 1.12 (1.01-1.24) |  | 1.64 (1.52-1.76) |  |

* Adjusted for age group, year and socioeconomic position.

RR = rate ratio; CI = confidence interval

Table S7. Crude and adjusted* associations between occupation category and mortality from ‘phthisis’ in Poisson regression (with overdispersion), excluding general labourers, the largest uncategorised occupation.

| Variable | | Crude Rate Ratio (95% CI) | p-value | Adjusted Rate Ratio (95% CI) | p-value |
| --- | --- | --- | --- | --- | --- |
| Occupation Category | Unexposed | Reference | <0.0001 | Reference | <0.0001 |
|  | Exposed | 1.13 (1.04-1.23) |  | 1.30 (1.23-1.39) |  |
|  | Uncategorised | 1.52 (1.42-1.63) |  | 1.62 (1.54-1.70) |  |

* Adjusted for age group, year and socioeconomic position.

RR = rate ratio; CI = confidence interval

Table S8. Crude and adjusted* associations between occupation category and mortality from ‘phthisis’ in Poisson regression (with overdispersion), stratified by denominator size.

| Tertile of denominator | Occupation category | Crude RR (95% CI) | Adjusted RR (95% CI) |
| --- | --- | --- | --- |
| 1 (smallest) | Little indoor contact | Reference | Reference |
|  | Indoor contact | 2.02 (1.51-2.71) | 1.47 (1.16-1.86) |
|  | Uncategorised | 1.60 (1.20-2.12) | 1.36 (1.08-1.72) |
|  |  |  |  |
| 2 | Little indoor contact | Reference | Reference |
|  | Indoor contact | 1.41 (1.12-1.77) | 1.62 (1.39-1.89) |
|  | Uncategorised | 1.52 (1.24-1.86) | 1.61 (1.40-1.85) |
|  |  |  |  |
| 3 | Little indoor contact | Reference | Reference |
|  | Indoor contact | 1.02 (0.87-1.18) | 1.26 (1.14-1.38) |
|  | Uncategorised | 1.67 (1.50-1.86) | 1.67 (1.55-1.80) |

* Adjusted for age group, year and socioeconomic position.

RR = rate ratio; CI = confidence interval

Table S9. Crude and adjusted* associations between occupation category and mortality from ‘phthisis’ in Poisson regression (with overdispersion), stratified by denominator size and excluding high risk groups

| Tertile of denominator | Occupation category | Crude RR (95% CI) | Adjusted RR (95% CI) |
| --- | --- | --- | --- |
| 1 (smallest) | Little indoor contact | Reference | Reference |
|  | Indoor contact | 1.67 (1.30-2.15) | 1.28 (1.05-1.55) |
|  | Uncategorised | 1.52 (1.19-1.93) | 1.33 (1.10-1.60) |
|  |  |  |  |
| 2 | Little indoor contact | Reference | Reference |
|  | Indoor contact | 1.42 (1.13-1.78) | 1.60 (1.37-1.87) |
|  | Uncategorised | 1.48 (1.22-1.79) | 1.54 (1.35-1.76) |
|  |  |  |  |
| 3 | Little indoor contact | Reference | Reference |
|  | Indoor contact | 1.05 (0.88-1.26) | 1.17 (1.04-1.33) |
|  | Uncategorised | 1.35 (1.20-1.53) | 1.51 (1.39-1.64) |

* Adjusted for age group, year and socioeconomic position.

RR = rate ratio; CI = confidence interval

Table S10: Crude and adjusted* associations between occupation category and mortality from ‘phthisis’ stratified by denominator size from random effects logistic regression.

| Tertile of denominator | Occupation category | Crude OR (95% CI) | Adjusted OR (95% CI) |
| --- | --- | --- | --- |
| 1 (smallest) | Little indoor contact | Reference | Reference |
|  | Indoor contact | 2.03 (1.39-2.98) | 1.65 (1.19-2.29) |
|  | Uncategorised | 1.51 (1.06-2.15) | 1.44 (1.06-1.95) |
|  |  |  |  |
| 2 | Little indoor contact | Reference | Reference |
|  | Indoor contact | 1.52 (1.10-2.09) | 1.51 (1.13-2.01) |
|  | Uncategorised | 1.49 (1.12-1.98) | 1.44 (1.11-1.86) |
|  |  |  |  |
| 3 | Little indoor contact | Reference | Reference |
|  | Indoor contact | 1.31 (0.90-1.90) | 1.62 (1.12-2.35) |
|  | Uncategorised | 1.44 (1.07-1.93) | 1.74 (1.29-2.35) |
|  |  |  |  |
| Overall | Little indoor contact | Reference | Reference |
|  | Indoor contact | 1.49 (1.18-1.90) | 1.69 (1.33-2.14) |
|  | Uncategorised | 1.50 (1.21-1.86) | 1.71 (1.39-2.11) |

* Adjusted for age group, year and socioeconomic position.

OR = odds ratio; CI = confidence interval

Table S11: Crude and adjusted* associations between occupation category and mortality from ‘phthisis’ based on negative binomial regression.

| Occupation category | Crude RR (95% CI) | Adjusted RR (95% CI)* |
| --- | --- | --- |
| Little indoor contact | Referent | Referent |
| Indoor contact | 1.58 (1.43-1.75) | 1.67 (1.55-1.80) |
| Uncategorised | 1.48 (1.36-1.61) | 1.62 (1.52-1.72) |

* Adjusted for age group, year and socioeconomic position.

RR = rate ratio; CI = confidence interval

Table S12: Crude and adjusted* associations between occupation category and mortality from ‘phthisis’ based on negative binomial regression, stratified by denominator size.

| Tertile of denominator | Occupation category | Crude RR (95% CI) | Adjusted RR (95% CI) |
| --- | --- | --- | --- |
| 1 (smallest) | Little indoor contact | Reference | Reference |
|  | Indoor contact | 2.26 (1.77-2.91) | 1.52 (1.23-1.88) |
|  | Uncategorised | 1.62 (1.28-20.6) | 1.30 (1.06-1.60) |
|  |  |  |  |
| 2 | Little indoor contact | Reference | Reference |
|  | Indoor contact | 1.50 (1.25-1.80) | 1.53 (1.34-1.75) |
|  | Uncategorised | 1.51 (1.29-1.77) | 1.50 (1.33-1.69) |
|  |  |  |  |
| 3 | Little indoor contact | Reference | Reference |
|  | Indoor contact | 1.15 (0.99-1.33) | 1.48 (1.34-1.64) |
|  | Uncategorised | 1.48 (1.33-1.65) | 1.73 (1.60-1.87) |

* Adjusted for age group, year and socioeconomic position.

RR = rate ratio; CI = confidence interval
